# Neuronal Activity Reporters as Drug Screening Platforms

**DOI:** 10.3390/mi13091500

**Published:** 2022-09-09

**Authors:** Igal Sterin, Ana C. Santos, Sungjin Park

**Affiliations:** 1 Department of Neurobiology, University of Utah School of Medicine, Salt Lake City, UT 84112, USA; 2 Center for Neuroscience, University of California, Davis, Davis, CA 95618, USA

**Keywords:** neuronal activity assay, live-cell assay, high-throughput drug screen, luminescence, fluorescence

## Abstract

Understanding how neuronal activity changes and detecting such changes in both normal and disease conditions is of fundamental importance to the field of neuroscience. Neuronal activity plays important roles in the formation and function of both synapses and circuits, and dysregulation of these processes has been linked to a number of debilitating diseases such as autism, schizophrenia, and epilepsy. Despite advances in our understanding of synapse biology and in how it is altered in disease, the development of therapeutics for these diseases has not advanced apace. Many neuronal activity assays have been developed over the years using a variety of platforms and approaches, but major limitations persist. Current assays, such as fluorescence indicators are not designed to monitor neuronal activity over a long time, they are typically low-throughput or lack sensitivity. These are major barriers to the development of new therapies, as drug screening needs to be both high-throughput to screen through libraries of compounds, and longitudinal to detect any effects that may emerge after continued application of the drug. This review will cover existing assays for measuring neuronal activity and highlight a live-cell assay recently developed. This assay can be performed with easily accessible lab equipment, is both scalable and longitudinal, and can be combined with most other established methods.

## 1. Introduction

Neuronal activity, including synaptic transmission and the activation of transcriptional programs, is critical for brain development and daily behaviors [1]. To understand how the activity of individual or sub-populations of neurons contributes to neuronal circuits, it is essential to monitor changes in neuronal activity. Our understanding of how neuronal activity is associated with transcriptional outputs and behaviors has advanced considerably owing to optogenetic and chemo-genetic tools that allow for the manipulation of genetically defined neuronal subsets. These tools have also helped elucidate the biology of neurons and synapses in healthy and disease states [2].

**Figure 1:**
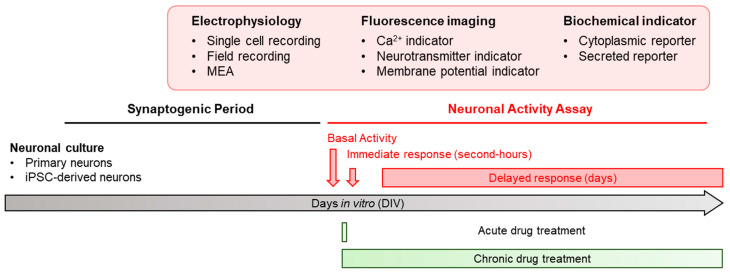
In vitro neuronal activity assay as drug screening platforms. Primary neurons derived from laboratory animals or induced pluripotent stem cell (iPSC)-derived human neurons are cultured and matured in vitro. After the synaptogenic period, neuronal activity can be measured by a variety of neuronal activity assays, including electrophysiological assays, fluorescence-based imaging, and/or biochemical assays. The acute and delayed effect of the drug treatment on neuronal activity can be monitored immediately or longitudinally for multiple days, respectively, depending on the invasiveness of assays and the capability of comparing the baseline activities over time.

Despite heavy research investment and biological advancements, developing therapeutics for neurological disorders remains challenging. For example, numerous genes have been associated with autism spectrum disorders and functional studies have illustrated their contributions to nervous system development [3]. However, targeted pharmacological therapies remain non-existent. This is especially concerning due to the heavy burden of neurological disorders on society [4]. The development of human induced pluripotent stem cells (iPSCs) and particularly patient-specific iPSC-derived neurons are a major advance not only as a physiological model for studying such diseases, but also by providing in vitro platforms for drug screening [5]. Many live-cell assays monitoring the changes in neuronal activity in vitro have been developed (Figure 1). However, the lack of robust, simple, and scalable assays for neuronal activity is a major obstacle for drug screening. A number of criteria are necessary to make a neuronal activity assay amenable to drug screens [6,7]. (i) It must be scalable and high-throughput such that diverse chemical compound libraries can be screened. (ii) It must be quantitative, so that the effect of such compounds can be detected and statistically tested. (iii) It should be as non-invasive as possible, to not disrupt cellular functions. (iv) It should be longitudinal, so that both acute and chronic drug effects can be identified. (v) Finally, it should be simple and economical as not to deter smaller groups from pursuing such investigations. For example, while some recently developed imaging techniques have addressed these requirements, sophisticated equipment and expertise is required, making them difficult to set up in a conventional laboratory setting. In this review, we will consider existing assays for neuronal activity using these criteria and highlight a recently developed neuronal activity reporter developed that addresses some shortcomings of previous methods [8] (Table 1).

## 2. Electrophysiological Recordings

Likely the longest standing method to measure neuronal activity, electrical recordings from a single neuron continues to be one of most thorough and precise approaches to the quantification of neuronal activity changes. In patch clamp, a glass pipette is placed on the cell surface to create a tight electrical seal between the pipette and the cell membrane [9]. Measuring electrical current and voltage changes across the membrane can reveal both presynaptic and postsynaptic characteristics and is highly suitable for detecting immediate effects of pharmacological treatments and other manipulations [10]. The resulting data can be of high quality and reproducibility, unfortunately, though, it is also a high skill technique with an expert being able to patch 15–30 cells per day. Patch-clamp automation systems have been developed but come with considerable caveats. They are difficult to optimize, may produce lower quality data, most are used with cell lines to study ion channel biology as opposed to action potentials in neurons, and only modestly increase the throughput of manual patch clamping [9,10,11] (for a detailed review of these technologies see [9]). These techniques have been used to characterize patient derived human neurons from iPSC, but they are not yet suited for high throughput drug screens [12]. Moreover, since the duration of the recording is limited by the viability of cells patched with an electrode, it cannot be used to monitor long-term changes in cellular physiology. This is an area to watch as the field continues to work to adapt this powerful assay to a high throughput format. 

Another form of electrical recordings are field recordings, where an electrode is placed extracellularly and can record population level electrical impulses as opposed to single cell membrane potential changes [13]. In most cases this is not advantageous because disease-causing cells are often near healthy cells making it computationally difficult to separate the signal from each cell. However, when large scale electrical disturbances are induced, as is the case in epilepsy models, they can be useful [14]. Unfortunately, even with the use of multiple electrophysiology recording rigs this remains a low to medium throughput approach. Combining multiple electrodes together constitutes a multi-electrode array (MEA), which can be used with multiple types of samples, such as primary neuron cultures or organoids. Although MEA can increase throughput and automation, they do not allow for recording from specific sub-populations of neurons, and require spike sorting and careful signal analysis [13]. As well, the density of electrodes does not allow for subcellular resolution and may misattribute single action potentials. However, in the last decade, commercially available high-density MEA (HD-MEA) have been fabricated that allow for subcellular resolution and higher temporal resolution, significantly increasing signal to noise and spike sorting to individual synapses and cellular compartments [15,16,17,18,19]. These devices have been used to map each synapse along an axon, track action potential progress along an axonal arbor, analyze whole network activity in culture over multiple days or organotypic slice, characterize disease models, and toxicity studies [16,17,19,20,21,22,23]. As a passive, noninvasive technique, HD-MEA can be paired with conventional endpoint assays and single cell omic analysis, as well as the genetic tools described below. This technology is clearly suited for high-throughput drug screening. Unfortunately, several technological hurdles have prevented HD-MEA from being employed in this capacity. Chief among them is fabricating these arrays into multi-well formats for high-throughput screening (HTS), the ability of neurons to grow on HD-MEA in these new formats, and the necessary data management and analysis systems necessary to sort mutli-day recordings from thousands of electrodes with microsecond temporal resolution [16,17,21,22]. If these hurdles are overcome, HD-MEA will be a valuable tool in drug screening for neurological therapies due to the multiple levels of analysis, non-invasiveness, and spatiotemporal resolution.

Another approach to combining multiple electrodes together consists of microelectrode probes, such as the Utah electrode array and more recently the neuropixel probes [24]. Since these microelectrode probes are most adequate for in vivo studies wherein a probe can be implanted into a specific brain region, they are considered more invasive and would require large numbers of animals. Although not practical for initial screening, this technique is being used to characterize compounds from in vitro screens. One of the issues of developing drugs for the nervous system is the complexity of how a drug will interact with other types of cells, as compared to the specific target cell(s) analyzed during screening [25]. By measuring how the electrical activity changes at the population level throughout the brain, these probes may more clearly reveal side-effects, such as a slow toxicity that may not be as apparent by animal behavior or phenotype [26]. Although there is much promise in the field of electrophysiological recordings for high throughput screening, the technical hurdles of reproducibility, scalability, and data management prevents its use in large scale drug screens.

## 3. Calcium and Neurotransmitter Indicators

Calcium is an important second messenger in many cell types, including neurons. Indeed, action potentials trigger a rise in intracellular calcium, that can be used as a measure of neuronal activity [27]. Genetically encoded calcium indicators have become widely used in recent years, the most prevalent of which is the GCaMP series. GCaMP is a fusion protein of calmodulin, the M13 myosin light chain kinase sequence, and a circularly permuted GFP (cpGFP) [27]. Upon binding cytoplasmic calcium, the excitation efficiency of the cpGFP increases, resulting in brighter fluorescence. The temporal resolution of GCaMPs has steadily increased, with the first generation able to reliably detect bursts of action potentials [28,29] and newer versions reportedly able to detect single action potentials triggered by electrical stimulation [28,30,31]. In practice though, the kinetics of GCaMPs limit them to suprathreshold events and a temporal resolution of 200–800 ms [28,32]. Neurotransmitter indicators work via a similar mechanism, but calmodulin is replaced by a domain, usually tethered to the membrane, that binds a specific neurotransmitter allowing for picomolar to micromolar detection thresholds depending on the neurotransmitter and temporal resolution ranging from tens to hundreds of milliseconds with a high dynamic range for glutamate and dopamine but much lower for other neurotransmitters [27,33]. These genetically encoded tools can be transgenes packaged into viral vectors, or induced by a recombinase, allowing for both temporal and spatial specificity. As well, they are being continuously improved upon by directed evolution for better sensitivity, brightness and kinetics [27,28,29,30,34,35,36,37]. 

Control and ALS patient derived human neurons expressing GCaMP6 were used to screen 1903 compounds and the authors functionally describe 3 of these [38]. These reporters can also be directed to distinct subcellular compartments by fusing a tag or protein to answer specific biological questions. Untagged GCaMPs measure cytosolic calcium which reports on burst activity sacrificing spatial resolution for increased signal [39,40]. A group recently used a glutamate indicator and calcium indicators targeted to either the postsynaptic compartment or the presynaptic compartment with electrical or pharmacological stimulation a in 96 well plates. Using self-fabricated plates and a custom microscope automation setup and a novel analysis pipeline, they were able to visualize calcium transients in aggregate and in individual spines, allowing for dissection of mechanisms of the few compounds tested [40]. Although their platform has not been used for HTS so far, they discuss the promise of this platform.

Zebrafish are particularly amenable to in vivo live imaging with genetically encoded indicators, due to being optically transparent, as well as high throughput drug screening due to their conservation of genes and pathways with mammals and ease of compound introduction and scalability [41,42,43,44,45]. For example, to find molecules that that effect dopamine neuron survival in a Parkinson’s disease model, 1043 bioactive molecules were screened (with 57 hits) in a zebrafish model using a dopamine indicator [46]. Whole brain imaging of larval zebrafish with GCaMP6 combined with machine learning was able to predict the therapeutic potential of a compound based on an initial known test library [47]. Recently, calcium imaging from free swimming zebrafish helped screen epilepsy drugs [48]. Calcium imaging in third instar fly larvae was also used to screen anticonvulsant drugs, although only a handful of compounds [49].

Most of the screens described required building and calibration of imaging automation equipment to correct unequal field illumination, and customized analysis software to deconvolute signal and resolution [50]. Moreover, as the reporter is consistently replenished by a constitutive promoter or the promoter activity can be affected by the compound added, it can be challenging to directly compare the baseline fluorescence intensity change over multiple imaging sessions following drug treatment. Hence, the imaging for such screens is usually carried out over one day, usually in one or two sessions total [38,40,46].

It is also notable that GCaMP transgenic animals display abnormalities, such as epileptiform activity [51]. Although the mechanism of these phenotypes has not been fully understood, GCaMPs may buffer cytoplasmic calcium and potentially cause biological changes [27,35]. Also, continued imaging sessions can lead to cellular phototoxicity [27,31,34,38,52]. As these tools are vital to the neuroscience community, they are constantly being optimized and assays for HTS may soon become much easier to use as automation becomes standardized and software is shared among the community. Although for now these tools lack some of the key characteristics of HTS, such as specialized equipment, issues with longitudinal studies and harm to the cells, they are still extremely powerful, especially in in vivo systems such as the fly and fish. 

## 4. Membrane Voltage Indicators:

Another class of genetically encoded reporters are fusions of the voltage sensing domains of different proteins to a fluorescent reporter [27]. Since they are genetically encoded fluorescent reporters, they have similar benefits and limitations as GCaMPs. Essentially, membrane depolarization causes a conformational shift in the voltage sensing domain, which changes the fluorescence of the attached reporter. These genetically encoded voltage indicators report directly on neuronal activity, as opposed to a proxy such as calcium or neurotransmitter release and some are able to detect subthreshold events that do not result in calcium release [53]. To detect these events, very fast kinetics of less than a microsecond is necessary, which creates a difficult tradeoff between temporal resolution and dynamic range [36,45,53,54]. They have been used both in culture and in living animals and can image entire networks of traveling action potentials [27]. There is large variability in the types of fusion proteins and their kinetics, and each needs to be carefully optimized. Some of these reporters work by using FRET donor-receptor pairs and their kinetics can resolve single action potentials [27]. They are, however, much more vulnerable than GCaMP to low signal to noise ratio, membrane localization, and photobleaching by high sampling rates. These assays continue to undergo further optimization [36], and are already having an impact on the field when combined with other assays. The ability to visualize slight changes in membrane potential as well as suprathreshold events holds a promise to find therapeutics for specific phenotypes. 

## 5. Immediate Early Genes

Immediate early genes are genes whose transcription is upregulated upon neuronal activation, which include *Arc/Arg3.1*, *c-fos*, and *Npas4*. These have been used as a readout of neuronal activity for many years. For example, transcripts of *Arc/Arg3.1* can be detected within minutes after a variety of neuronal stimulations [55,56]. Immunostaining for these proteins has been used to create maps of neuronal activation after various behavioral tasks [57,58]. The specific network of neurons activated by these behavioral tasks is termed an ensemble. By mapping the expression of IEGs and using them to drive reporter expression, these ensembles can be reliably labeled [59]. Expression of an optogenetic or chemogenetic tool allows for selectively manipulating the activity of neurons in an ensemble whereas driving a synaptic tag with a photo reversible fluorophore can allow for spatial and temporal neuronal activation information. These results support that the promoter activity of immediate early genes reliably reflect neuronal activity in vivo. While there is much excitement about these tools to help understand learning and memory, they seem to have little application to drug screening. However, regulatory elements of an immediate early gene can be coupled with a biochemical reporter such as a luciferase, allowing for continued monitoring of expression over time [60]. By combining a regulatory region of the *Arc/Arg3.1* gene with a secreted *Gaussia* luciferase (Gluc), we created a neuronal activity reporter which we have named secreted neuronal activity reporter (SNAR) [8].

## 6. SNAR

**Figure 2:**
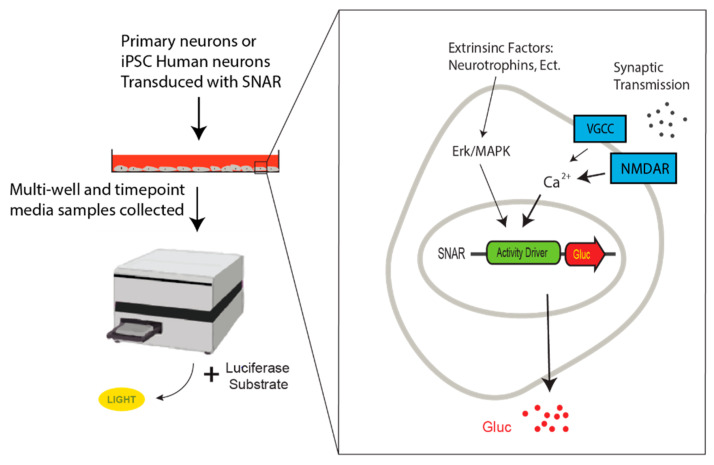
Schematic of secreted neuronal activity reporter (SNAR) assay. Human iPSC-derived neurons or primary neuron cultures are transduced with SNAR. Media samples are collected at multiple time points and luciferase activity is recorded after the addition of the Coelenterazine, the *Gaussia* luciferase substrate by a luminometer. SNAR is calcium dependent and predominantly induced by NMDA receptor activation. Voltage gated calcium channels (VGCC) as well as the Erk/MAPK pathway also contribute to SNAR, albeit to a lower extent.

To generate an activity-dependent, live-cell assay, we used Gluc [61], which offers significant advantages: (i) it is a small protein (19 kDa) allowing for easy packaging into viral vectors; (ii) it is naturally secreted upon synthesis, allowing for continued monitoring of the reporter by sampling the cell culture media over time; (iii) it is 1000 fold brighter than the more commonly used *Firefly* and *Renilla* luciferases making it extremely sensitive. Gluc has been used as a reporter for monitoring other biological systems, such as a tumor model [62] and more recently the dynamics of Arc/Arg3.1 translation [63]. SNAR consists of a construct where Gluc is driven by a promoter constructed of several repeats of a regulatory region of the *Arc/Arg3.1* gene previously shown to respond to synaptic activity [64] (Figure 2). 

Consistent with other studies on the regulation of endogenous *Arc/Arg3.1*, SNAR dynamically responds to various manipulations of neuronal activity (Figure 1). Blockage of neuronal firing, NMDA receptor transmission, or voltage-gated calcium channels suppress SNAR activity. On the other hand, inhibition of GABA receptor transmission or the application of either astrocyte-conditioned medium or BNDF induces SNAR activity. The SNAR assay reliably detects changes in neuronal activity caused by epilepsy drugs, further demonstrating its application as a drug screening tool. This assay is ideal for drug screening as media sampling can be automated, allowing easy scalability. Similarly, it requires no specialized equipment, and has minimal impact on the biology of the cell as Gluc is secreted after being produced. Reliable measurements can be made from as little as one microliter of media, allowing for repeated sampling of between 5 and 20 microliters without replenishing the media, although multiple time points within the same day may disturb the culture if it is repeatedly withdrawn from the incubator. In addition, this assay can be combined with any of the assays listed above, optogenetic and chemogenetic tools, other imaging-based approaches, and conventional endpoint assays. It can also be applied to patient-derived iPSC neurons to characterize neuronal activity defect in the disease state and simultaneously screen the drugs that reverse it. Thus, it can potentially be used to screen for personalized medicines for specific neurological disorders such as drug-resistant epilepsy. By using Cre-recombinase specific expression, we can screen for drugs that specifically change a subpopulation of neurons. It is notable that different immediate early genes show varied temporal kinetics and can be induce by distinct upstream signaling [56,65,66]. Since SNAR is monitoring the activity of Arc regulatory elements, combining SNAR with other immediate early gene reporters will provide a broader neuronal activation profile. 

The use of Gluc as a reporter makes SNAR extremely simple and sensitive, however there are some limitations that should be taken into consideration. Due to the ongoing release of luciferase that was already synthesized, there is a lag-time, limiting the time-resolution of the assay. Thus, SNAR is not ideal for detecting immediate neuronal responses. Since Gluc is secreted, SNAR does not provide spatial resolution. However, this can be addressed by conventional imaging techniques or inducing cell-type specific expression of SNAR by a recombinase. Similar to other genetically encoded reporters, the absolute level of the SNAR reporter can be variable depending on the culture conditions and infection rate. Thus, normalization of the SNAR signal to a pre-treatment state or a control reporter is critical. Since SNAR is based on multiple quantitative samples from the same neuronal population, a paired analysis of the same neurons before and after the manipulation greatly improves the consistency of the assay. 

## 7. Conclusions

Developing therapeutics for neurological disorders has lagged behind our understanding of synapse and neuronal biology. This can be partially attributed to the lack of an optimal neuronal activity assay for high-throughput drug screening. In this review, we compared the available assays for neuronal activity with an assay recently developed in our lab for their practicality in drug screening (Table 1). Although each of the established assays-electrophysiology, calcium, voltage and neurotransmitter indicators, and immediate early genes-can monitor neuronal activity, none of them are ideal for large scale drug screening due to throughput, technical considerations, or difficulties optimizing conditions across multiple sessions. SNAR, based on a secreted luciferase driven by a neuronal activity promoter, is ideal for large scale initial drug screening because of its intra-assay reliability, longitudinal tracing, ease of use, and quantitative nature.

## Figures and Tables

**Table 1:** Comparing neuronal activity assays for their applicability to high-throughput drug screening in vitro.

	Patch-Clamp Recordings	Microelectrode Arrays (MEA)	Genetically Encoded Calcium Indicators	Genetically Encoded Neurotransmitter Indicators	Genetically Encoded Voltage Indicators	SNAR
Scalability	Low	MEA: medium-high High density-MEA: Low	Medium-high: automation of imaging: 96 wells possible	Medium-high: automation of imaging: 96 wells possible	Low: need to optimize each tool for each new screen	Very high, the whole assay can be automated
Dynamic Range, Signal to noise ratio	Very high	Very high	High, continue increasing	Glutamate and dopamine: High Others: low-medium	Low	Very high: luciferases are linearly quantitative
Biological disruption	Very high: cells usually die afterward	Minimal, more from high density	Low, some cytotoxic effects	Low	Low	Very low: the reporter is secreted
Longitudinal	Low: see above	Extremely high if culture survives plating on electrodes	Medium, some care needs to be taken comparing between days	Medium, some care needs to be taken comparing between days	Medium	Extremely high: can be followed for hours, days, or weeks
Simplicity	Skill, time, and equipment intensive	Complicated to manufacture	Variable: automated imaging, optimization of indicator	Variable: automated imaging, optimization of indicator	Complicated: tools are being optimized	Easy to use, simple lab equipment, easy controls, and optimization
Temporal resolution	<1 ms	<1 ms	200–800 ms	10–800 ms	<1 ms–200 ms	30 min
Spatial scale	Whole-cell to axon	Network to synapse	Brain to synapse	Brain to synapse	Brain to synapse	Network
Computational requirements	Low-medium with established software	High: spike sorting and noise deconvolution: new technology	Medium-high depending on screen	Medium-high depending on screen	Medium-high depending on screen	Very low

## References

[1] Eminatohara K., Eakiyoshi M., Eokuno H. (2016). Role of Immediate-Early Genes in Synaptic Plasticity and Neuronal Ensembles Underlying the Memory Trace. Front. Mol. Neurosci..

[2] DeNardo L., Luo L. (2017). Genetic strategies to access activated neurons. Curr. Opin. Neurobiol..

[3] Parenti I., Rabaneda L.G., Schoen H., Novarino G. (2020). Neurodevelopmental Disorders: From Genetics to Functional Pathways. Trends Neurosci..

[4] Carroll W.M. (2019). The global burden of neurological disorders. Lancet Neurol..

[5] Fink J.J., Levine E.S. (2018). Uncovering True Cellular Phenotypes: Using Induced Pluripotent Stem Cell-Derived Neurons to Study Early Insults in Neurodevelopmental Disorders. Front. Neurol..

[6] Nierode G., Kwon P.S., Dordick J.S., Kwon S.-J. (2016). Cell-Based Assay Design for High-Content Screening of Drug Candidates. J. Microbiol. Biotechnol..

[7] Wang L., Yu C., Wang J. (2019). Development of reporter gene assays to determine the bioactivity of biopharmaceuticals. Biotechnol. Adv..

[8] Santos A.C., Chiola S., Yang G., Shcheglovitov A., Park S. (2021). Secreted Reporter Assay Enables Quantitative and Longitudinal Monitoring of Neuronal Activity. Eneuro.

[9] Liu C., Li T., Chen J. (2019). Role of High-Throughput Electrophysiology in Drug Discovery. Curr. Protoc. Pharmacol..

[10] Noguchi A., Ikegaya Y., Matsumoto N. (2021). In Vivo Whole-Cell Patch-Clamp Methods: Recent Technical Progress and Future Perspectives. Sensors.

[11] Obergrussberger A., Brüggemann A., Goetze T.A., Rapedius M., Haarmann C., Rinke I., Becker N., Oka T., Ohtsuki A., Stengel T. (2016). Automated Patch Clamp Meets High-Throughput Screening: 384 Cells Recorded in Parallel on a Planar Patch Clamp Module. J. Lab. Autom..

[12] Rosholm K.R., Badone B., Karatsiompani S., Nagy D., Seibertz F., Voigt N., Bell D.C. (2022). Adventures and Advances in Time Travel with Induced Pluripotent Stem Cells and Automated Patch Clamp. Front. Mol. Neurosci..

[13] Harris K.D., Quiroga R.Q., Freeman J., Smith S.L. (2016). Improving data quality in neuronal population recordings. Nat. Neurosci..

[14] Barker-Haliski M.L., Johnson K., Billingsley P., Huff J., Handy L.J., Khaleel R., Lu Z., Mau M.J., Pruess T.H., Rueda C. (2017). Validation of a Preclinical Drug Screening Platform for Pharmacoresistant Epilepsy. Neurochem. Res..

[15] Shabestari P.S., Buccino A.P., Kumar S.S., Pedrocchi A., Hierlemann A. (2021). A modulated template-matching approach to improve spike sorting of bursting neurons. IEEE Biomed. Circuits Syst. Conf..

[16] Müller J., Ballini M., Livi P., Chen Y., Radivojevic M., Shadmani A., Viswam V., Jones I.L., Fiscella M., Diggelmann R. (2015). High-resolution CMOS MEA platform to study neurons at subcellular, cellular, and network levels. Lab Chip.

[17] Lonardoni D., Amin H., Zordan S., Boi F., Lecomte A., Angotzi G.N., Berdondini L. (2019). Active High-Density Electrode Arrays: Technology and Applications in Neuronal Cell Cultures. Adv. Neurobiol..

[18] Liu Y., Li X., Chen J., Yuan C. (2020). Micro/Nano Electrode Array Sensors: Advances in Fabrication and Emerging Applications in Bioanalysis. Front. Chem..

[19] Kim J., Shin H., Kweon S.-J., Lee S., Ha S., Je M. (2021). A Scalable Readout IC Based on Wideband Noise Cancelling for Full-Rate Scanning of High-Density Microelectrode Arrays. Annu. Int. Conf. IEEE Eng. Med. Biol. Soc..

[20] Vassallo A., Chiappalone M., Lopes R.D.C., Scelfo B., Novellino A., Defranchi E., Palosaari T., Weisschu T., Ramirez T., Martinoia S. (2016). A multi-laboratory evaluation of microelectrode array-based measurements of neural network activity for acute neurotoxicity testing. NeuroToxicology.

[21] Maccione A., Garofalo M., Nieus T., Tedesco M., Berdondini L., Martinoia S. (2012). Multiscale functional connectivity estimation on low-density neuronal cultures recorded by high-density CMOS Micro Electrode Arrays. J. Neurosci. Methods.

[22] Emmenegger V., Obien M.E.J., Franke F., Hierlemann A. (2019). Technologies to Study Action Potential Propagation With a Focus on HD-MEAs. Front. Cell. Neurosci..

[23] Buccino A.P., Yuan X., Emmenegger V., Xue X., Gänswein T., Hierlemann A. (2022). An automated method for precise axon reconstruction from recordings of high-density micro-electrode arrays. J. Neural Eng..

[24] Steinmetz N.A., Aydin C., Lebedeva A., Okun M., Pachitariu M., Bauza M., Beau M., Bhagat J., Böhm C., Broux M. (2021). Neuropixels 2.0: A miniaturized high-density probe for stable, long-term brain recordings. Science.

[25] Ahfeldt T., Litterman N.K., Rubin L.L. (2016). Studying human disease using human neurons. Brain Res..

[26] Ratner M.H., Farb D.H. (2022). Probing the Neural Circuitry Targets of Neurotoxicants In Vivo through High Density Silicon Probe Brain Implants. Front. Toxicol..

[27] Broussard G., Liang R., Etian L. (2014). Monitoring activity in neural circuits with genetically encoded indicators. Front. Mol. Neurosci..

[28] Podor B., Hu Y.-L., Ohkura M., Nakai J., Croll R., Fine A. (2015). Comparison of genetically encoded calcium indicators for monitoring action potentials in mammalian brain by two-photon excitation fluorescence microscopy. Neurophotonics.

[29] Akerboom J., Chen T.-W., Wardill T., Tian L., Marvin J., Mutlu S., Calderón N.C., Esposti F., Borghuis B.G., Sun X.R. (2012). Optimization of a GCaMP Calcium Indicator for Neural Activity Imaging. J. Neurosci..

[30] Ohkura M., Sasaki T., Sadakari J., Gengyo-Ando K., Kagawa-Nagamura Y., Kobayashi C., Ikegaya Y., Nakai J. (2012). Genetically Encoded Green Fluorescent Ca^2+^ Indicators with Improved Detectability for Neuronal Ca^2+^ Signals. PLoS ONE.

[31] Chen T.-W., Wardill T.J., Sun Y., Pulver S.R., Renninger S.L., Baohan A., Schreiter E.R., Kerr R.A., Orger M.B., Jayaraman V. (2013). Ultrasensitive fluorescent proteins for imaging neuronal activity. Nature.

[32] Xiao D., Vanni M.P., Mitelut C.C., Chan A.W., LeDue J.M., Xie Y., Chen A.C., Swindale N.V., Murphy T.H. (2017). Mapping cortical mesoscopic networks of single spiking cortical or sub-cortical neurons. eLife.

[33] Leopold A., Shcherbakova D.M., Verkhusha V.V. (2019). Fluorescent Biosensors for Neurotransmission and Neuromodulation: Engineering and Applications. Front. Cell. Neurosci..

[34] Wu N., Nishioka W.K., Derecki N.C., Maher M.P. (2019). High-throughput-compatible assays using a genetically-encoded calcium indicator. Sci. Rep..

[35] Tian L., Hires S.A., Looger L.L. (2012). Imaging Neuronal Activity with Genetically Encoded Calcium Indicators. Cold Spring Harb. Protoc..

[36] St-Pierre F., Chavarha M., Lin M.Z. (2015). Designs and sensing mechanisms of genetically encoded fluorescent voltage indicators. Curr. Opin. Chem. Biol..

[37] Lin M.Z., Schnitzer M.J. (2016). Genetically encoded indicators of neuronal activity. Nat. Neurosci..

[38] Boivin B., Roet K.C.D., Huang X., Karhohs K.W., Rohban M.H., Sandoe J., Wiskow O., Maeda R., Grantham A., Dornon M.K. (2022). A multiparametric activity profiling platform for neuron disease phenotyping and drug screening. Mol. Biol. Cell.

[39] Verschuuren M., Verstraelen P., Barriga G.G.-D., Cilissen I., Coninx E., Verslegers M., Larsen P.H., Nuydens R., De Vos W.H. (2019). High-throughput microscopy exposes a pharmacological window in which dual leucine zipper kinase inhibition preserves neuronal network connectivity. Acta Neuropathol. Commun..

[40] Van Dyck M., Mishra R.K., Pestana F., Verstraelen P., Lavreysen H., Pita-Almenar J.D., Kashikar N.D., De Vos W.H. (2021). High-throughput Analysis of Synaptic Activity in Electrically Stimulated Neuronal Cultures. Neuroinformatics.

[41] Muto A., Ohkura M., Abe G., Nakai J., Kawakami K. (2013). Real-Time Visualization of Neuronal Activity during Perception. Curr. Biol..

[42] Walker A.S., Burrone J., Meyer M.P. (2013). Functional imaging in the zebrafish retinotectal system using RGECO. Front. Neural Circuits.

[43] Zhang T., Peterson R.T., Cartner S.C., Eisen J.S., Farmer S.C., Guillemin K.J., Kent M.L., Sanders G.E. (2020). Chapter 51—Zebrafish as a Platform for Drug Screening. The Zebrafish in Biomedical Research.

[44] Strange K. (2016). Drug Discovery in Fish, Flies, and Worms. ILAR J..

[45] Potekhina E.S., Bass D.Y., Kelmanson I.V., Fetisova E.S., Ivanenko A.V., Belousov V.V., Bilan D.S. (2020). Drug Screening with Genetically Encoded Fluorescent Sensors: Today and Tomorrow. Int. J. Mol. Sci..

[46] Kim G.-H.J., Mo H., Liu H., Okorie M., Chen S., Zheng J., Li H., Arkin M., Huang B., Guo S. (2022). In Vivo Dopamine Neuron Imaging-Based Small Molecule Screen Identifies Novel Neuroprotective Compounds and Targets. Front. Pharmacol..

[47] Lin X., Duan X., Jacobs C., Ullmann J., Chan C.-Y., Chen S., Cheng S.-H., Zhao W.-N., Poduri A., Wang X. (2018). High-throughput brain activity mapping and machine learning as a foundation for systems neuropharmacology. Nat. Commun..

[48] Kanyo R., Wang C.K., Locskai L.F., Li J., Allison W.T., Kurata H.T. (2020). Functional and behavioral signatures of Kv7 activator drug subtypes. Epilepsia.

[49] Streit A.K., Fan Y.N., Masullo L., Baines R.A. (2016). Calcium Imaging of Neuronal Activity in Drosophila Can Identify Anticonvulsive Compounds. PLoS ONE.

[50] Xue Y. (2022). Computational optics for high-throughput imaging of neural activity. Neurophotonics.

[51] Steinmetz N.A., Buetfering C., Lecoq J., Lee C.R., Peters A.J., Jacobs E., Coen P., Ollerenshaw D.R., Valley M.T., de Vries S. (2017). Aberrant Cortical Activity in Multiple GCaMP6-Expressing Transgenic Mouse Lines. eNeuro.

[52] Larsch J., Ventimiglia D., Bargmann C.I., Albrecht D.R. (2013). High-throughput imaging of neuronal activity in *Caenorhabditis elegans*. Proc. Natl. Acad. Sci. USA.

[53] Chen Z., Truong T.M., Ai H.-W. (2017). Illuminating Brain Activities with Fluorescent Protein-Based Biosensors. Chemosensors.

[54] Nakajima R., Jung A., Yoon B.-J., Baker B.J. (2016). Optogenetic Monitoring of Synaptic Activity with Genetically Encoded Voltage Indicators. Front. Synaptic Neurosci..

[55] Guzowski J.F., McNaughton B.L., Barnes C.A., Worley P.F. (1999). Environment-specific expression of the immediate-early gene Arc in hippocampal neuronal ensembles. Nat. Neurosci..

[56] Tyssowski K., DeStefino N.R., Cho J.-H., Dunn C.J., Poston R.G., Carty C.E., Jones R.D., Chang S.M., Romeo P., Wurzelmann M.K. (2018). Different Neuronal Activity Patterns Induce Different Gene Expression Programs. Neuron.

[57] Daberkow D.P., Riedy M.D., Kesner R.P., Keefe K.A. (2007). Arc mRNA induction in striatal efferent neurons associated with response learning. Eur. J. Neurosci..

[58] Ivanova T., Matthews A., Gross C., Mappus R., Gollnick C., Swanson A., Bassell G., Liu R. (2011). Arc/Arg3.1 mRNA expression reveals a subcellular trace of prior sound exposure in adult primary auditory cortex. Neuroscience.

[59] Sørensen A.T., Cooper Y.A., Baratta M.V., Weng F.-J., Zhang Y., Ramamoorthi K., Fropf R., LaVerriere E., Xue J., Young A. (2016). A robust activity marking system for exploring active neuronal ensembles. eLife.

[60] Kawashima T., Kitamura K., Suzuki K., Nonaka M., Kamijo S., Takemoto-Kimura S., Kano M., Okuno H., Ohki K., Bito H. (2013). Functional labeling of neurons and their projections using the synthetic activity–dependent promoter E-SARE. Nat. Methods.

[61] Shao N., Bock R. (2008). A codon-optimized luciferase from Gaussia princeps facilitates the in vivo monitoring of gene expression in the model alga Chlamydomonas reinhardtii. Curr. Genet..

[62] Badr C.E., Niers J.M., Tjon-Kon-Fat L.-A., Noske D.P., Wurdinger T., Tannous B.A. (2009). Real-time monitoring of nuclear factor kappaB activity in cultured cells and in animal models. Mol. Imaging.

[63] Na Y., Park S., Lee C., Kim D.-K., Park J.M., Sockanathan S., Huganir R.L., Worley P.F. (2016). Real-Time Imaging Reveals Properties of Glutamate-Induced Arc/Arg 3.1 Translation in Neuronal Dendrites. Neuron.

[64] Inoue M., Yagishita-Kyo N., Nonaka M., Kawashima T., Okuno H., Bito H. (2010). Synaptic Activity Responsive Element (SARE). Commun. Integr. Biol..

[65] Sheng H.Z., Fields R.D., Nelson P.G. (1993). Specific regulation of immediate early genes by patterned neuronal activity. J. Neurosci. Res..

[66] Neumann-Haefelin T., Wießner C., Vogel P., Back T., Hossmann K.-A. (1994). Differential Expression of the Immediate Early Genes c-*Fos*, c-*Jun, Jun* B, and *NGFI*-B in the Rat Brain following Transient Forebrain Ischemia. J. Cereb. Blood Flow Metab..

